# Influence of Carbon Nanotube Addition on Microstructure and Microwave Heating Performance of Polycarbosilane-Based Silicon Carbide

**DOI:** 10.3390/ma18071454

**Published:** 2025-03-25

**Authors:** Chang-Hun Hwang, Jong-Ha Beak, Se-Yun Kim

**Affiliations:** 1 Department of Advanced Materials Science and Enginnering, Kyungnam University, Changwon 51767, Republic of Korea; 2 Department of Mechatronics Engineering, Kyungnam University, Changwon 51767, Republic of Korea

**Keywords:** silicon carbide (SiC), polycarbosilane (PCS), microwave heating, ultrahigh-temperature heating, carbon nanotube (CNT)

## Abstract

The microwave heating of silicon carbide is induced at a specific frequency of 2.45 GHz, leading to rapid heating within a temperature range of several hundred degrees Celsius. In this study, a mechanochemical curing process using iodine was employed to cure polycarbosilane (PCS), followed by the addition of carbon nanotubes (CNTs) to produce mixed polymer powders. The effects of the CNT addition on the microstructure, crystalline structure, and microwave heating properties were investigated. The findings indicated that the incorporation of CNTs generally led to a reduction in the number of micropores; however, when the CNT concentration exceeded 10 wt%, the aggregation of CNTs became evident. In terms of microwave heating properties, the sample containing 0.1 wt% CNTs achieved the highest temperature, whereas samples with a higher CNT content demonstrated a heating limit of approximately 500 °C. Remarkably, post-processing of the specimens with 10 wt% CNTs enabled rapid heating to approximately 1800 °C within 4 s of microwave exposure.

## 1. Introduction

Silicon carbide (SiC) exhibits excellent mechanical strength, high-temperature stability, and chemical resistance, making it suitable for several applications that require lightweight and high-performance characteristics in the aerospace and defense industries [1,2,3]. SiC can be synthesized through multiple processes, among which SiC fibers derived from polycarbosilane (PCS) precursors serve as representative reinforcement material for ceramic matrix composites [4,5]. Notably, its rapid heating behavior upon exposure to a specific microwave frequency (2.45 GHz) suggests the existence of diverse potential applications that leverage this characteristic [6,7]. The study of the fabrication of microwave heating materials based on polycarbosilane was introduced by Joo [8], who investigated the microwave heating characteristics by manufacturing fibers and blocks. The study reported differences in heating temperature and thermal generation behavior depending on the specimen’s shape. Additionally, Khishigbayar [9] introduced a study of the fabrication of silicon carbide fiber sheets and examined their microwave heating properties.

The organometallic polymer PCS is widely recognized as a representative precursor for the fabrication of SiC ceramics. Generally, PCS powder can be molded or dissolved in a solution, followed by curing and pyrolysis to produce SiC ceramic materials [10,11,12]. This approach facilitates the application of SiC materials in different fields by enhancing their processability.

Recently, extensive research has been conducted on the microwave heating performance of SiC materials fabricated using PCS, and our research team has previously conducted related preliminary studies. It has been observed that SiC, synthesized under varying oxidation curing and pyrolysis conditions of PCS, exhibits changes in microwave heating performance depending on the microstructural and crystallographic transformations [13]. Subsequently, a mechanochemical curing process utilizing iodine was developed to replace the oxidation curing process. Investigating the microwave heating performance of SiC, produced using PCS powder and cured using this method, revealed differences in the microstructure depending on the amount of iodine added, which in turn influenced the microwave heating performance [14].

Previous research has confirmed that microstructural changes influence microwave heating performance. Based on these findings, additional studies have been conducted to form a secondary phase by incorporating additives. Additive selection is focused on materials that can retain their structural integrity during high-temperature pyrolysis without undergoing vaporization, melting, or deformation. Among various candidate materials, carbon nanotubes (CNTs) have been identified as chemically inert under high-temperature inert atmospheres and are compatible with the PCS composition. Accordingly, CNTs were introduced as additives and their effects on microwave heating performance were investigated [15,16].

In this study, PCS-based SiC was synthesized by subjecting PCS powder to iodine mechanochemical curing followed by CNT addition. Iodine vapor curing is a process that utilizes the characteristic of iodine (I) to vaporize at relatively low temperatures to cure polycarbosilane [17,18,19]. However, the inhalation of iodine vapor through the respiratory system can be hazardous to the bronchial passages, and there are concerns regarding the chemical contamination of equipment used in the iodine vapor curing process [20,21]. Therefore, a mechanical curing process was selected to prevent vaporization [22]. The effects of this addition on the microstructure, crystalline structure, and microwave-induced heating performance of PCS-derived SiC were systematically investigated. Notably, depending on the specimen geometry, an ultrahigh-temperature rapid heating phenomenon was observed.

## 2. Materials and Methods

PCS (Daeho I&T, Changwon, Republic of Korea) was used as the precursor, with the addition of 0.25 wt% iodine (Thermo Scientific, Waltham, MA, USA), followed by mechanochemical curing facilitated by alumina-induced reactions. After curing, CNTs were added to the PCS at weight fractions of 0.1, 1, and 10 wt%, and then placed in a vial and shaken. The resulting powder mixtures were introduced into a 35 mm molding die and pressed under 5 tons for 3 min to form compacts. The green body samples formed were subsequently pyrolyzed at 1300 °C for 3 h in an argon atmosphere to fabricate silicon carbide (SiC) microwave heating samples.

Among the pyrolyzed specimens, those containing 10 wt% CNTs underwent four different post-processing treatments. The first method involved polishing the specimen surface using 1000-grit sandpaper. The second method involved fracturing and reconnecting the specimen at its midpoint. The third method involved perforating the center of the disk-shaped specimen. The fourth method involved the removal of the central perforation in an arbitrary direction using a high-speed cutter.

The microwave heating performance test was conducted using a microwave generator (Daeho I&T, Republic of Korea), where the specimens were exposed to 2.45 GHz microwaves at a power output of 1.85 kW for 60 min. The heating behavior of the specimens was recorded in real time using an infrared thermal imaging camera (PI1M, OPTRIS, Berlin, Germany) with a detection range of 400–1800 °C.

The fracture surface microstructure and composition of the fabricated specimens were analyzed using a field-emission scanning electron microscope equipped with a heating stage (Apreo S, Thermo Fisher Scientific, USA). The crystal structures were characterized using X-ray diffraction (XRD) (SmartLab SE, 40 kV/50 mA, Rigaku Corporation, Tokyo, Japan).

## 3. Results

**Figure 1:**
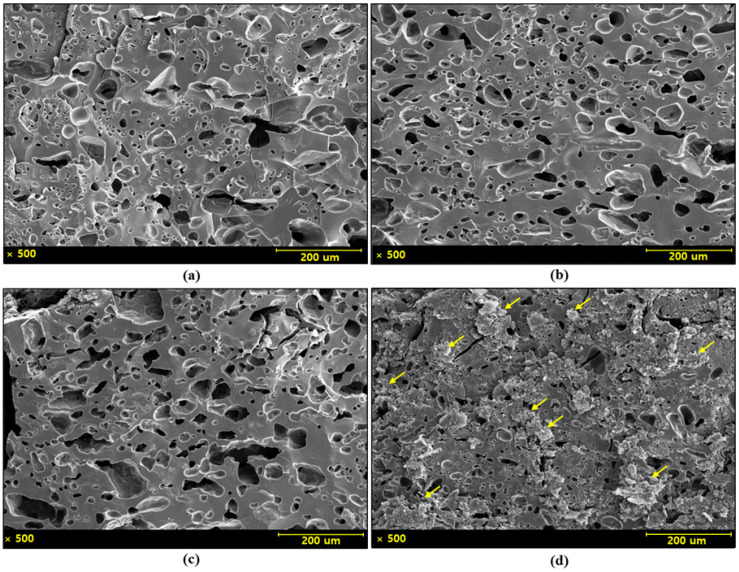
Microstructure images of PCS-derived SiC with varying CNT contents: (**a**) without CNT, (**b**) 0.1 wt% CNT, (**c**) 1 wt% CNT, and (**d**) 10 wt% CNT (yellow arrows indicate secondary phase-like microstructures).

The effects of CNT addition on the microstructure of SiC, synthesized via PCS, pyrolysis were investigated. Specimens were fabricated by pyrolyzing PCS in an argon atmosphere at 1300 °C for 3 h and their microstructures were analyzed, as shown in Figure 1. The microstructure of the specimen without CNT exhibits a wide distribution of differently sized pores. In contrast, the addition of 0.1 wt% and 1 wt% CNTs led to a reduction in microporosity. However, in the specimen containing 10 wt% CNTs, a secondary phase-like microstructure was observed, although overall porosity was reduced.

**Figure 2:**
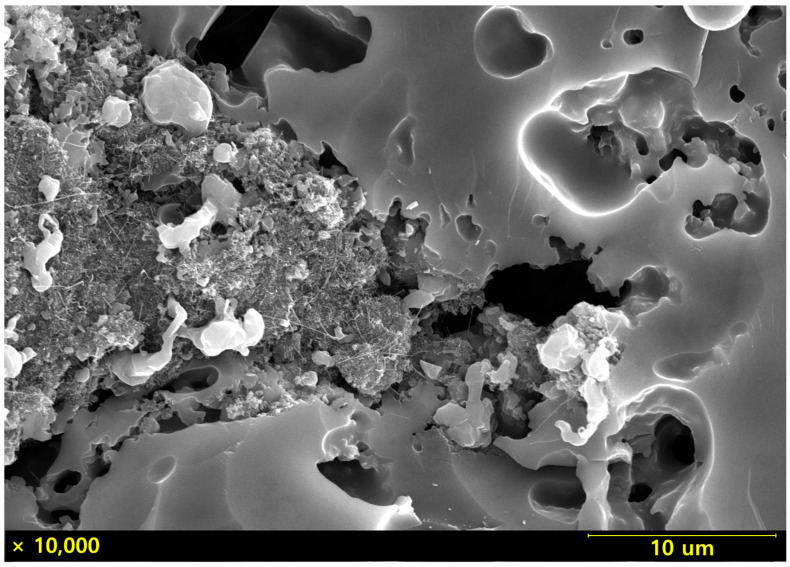
Microstructure images of SiC fabricated with 10 wt% CNT addition.

**Figure 3:**
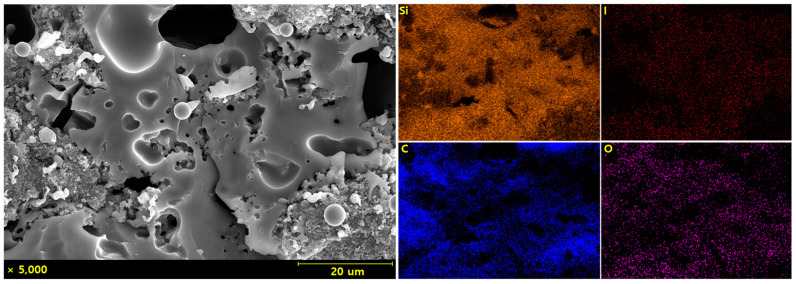
Mapping analysis of SiC fabricated with 10 wt% CNT addition.

A high-magnification analysis of the secondary phase-like regions in the microstructure shown in Figure 1d is presented in Figure 2. The regions suspected to be secondary phases exhibited aggregated fibrous structures, which were inferred to result from the agglomeration of CNTs that were not sufficiently dispersed during the powder-mixing process and the amorphous phase formed during pyrolysis. Mapping analysis of this region was conducted, and the results are shown in Figure 3. The high carbon content detected in these areas further supports the conclusion that the aggregates observed were CNT clusters.

**Figure 4:**
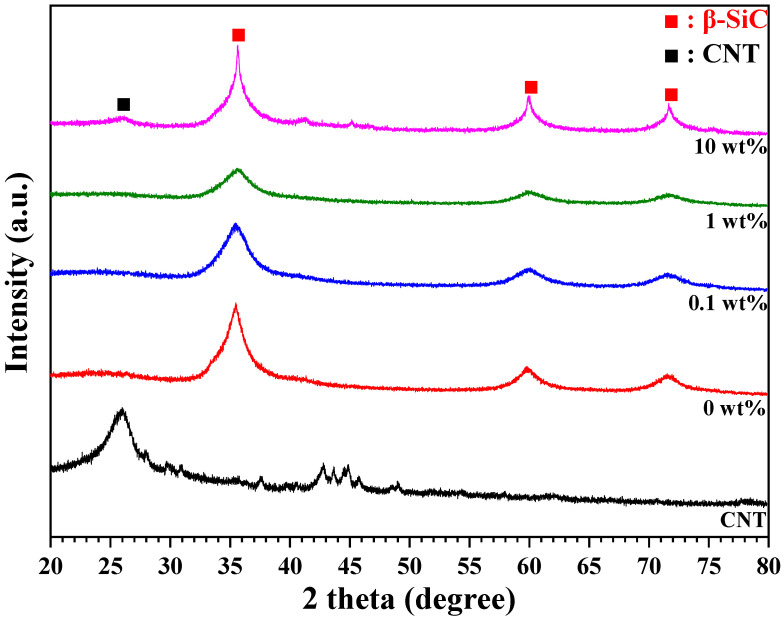
Crystal structure of PCS-derived SiC with varying CNT contents.

To investigate the effect of CNT content on the crystalline structure, XRD analysis was conducted, and the results are presented in Figure 4. When 0.1 wt% and 1 wt% CNTs were added, no additional crystalline patterns were detected; however, the intensity of the β-SiC peaks decreased as the CNT content increased. In contrast, when 10 wt% CNTs were added, the intensity of the β-SiC peaks increased, with some CNT peaks detected. This phenomenon is attributed to the residual CNT agglomeration shown in Figure 3. The Scherrer equation was applied to the XRD results to determine the crystallite size of β-SiC. The crystallite sizes obtained were 3.43, 3.01, 2.91, and 4.20 nm for specimens without CNTs and with CNT contents of 0.1, 1, and 10 wt%, respectively.

**Figure 5:**
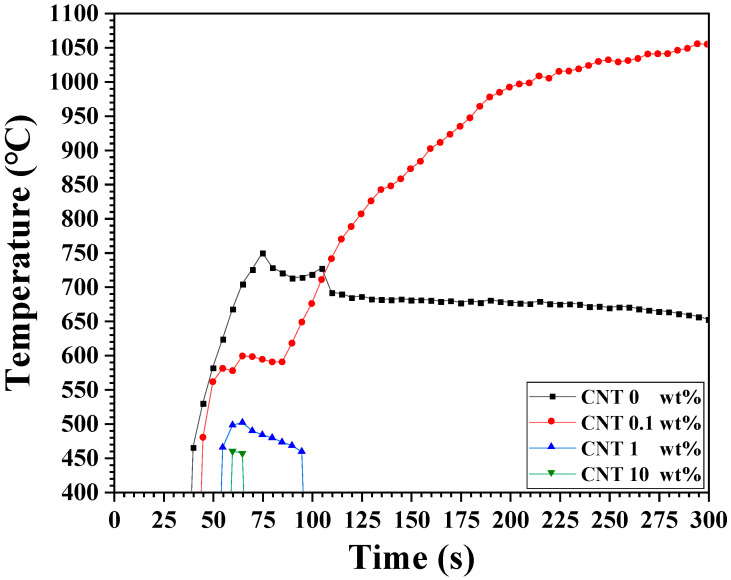
Microwave heating performance of specimens fabricated with varying CNT contents.

The microwave heating characteristics of SiC fabricated with CNTs were investigated and the results are presented in Figure 5. The specimen without CNT reached a microwave heating temperature of 749 °C at 300 s. By contrast, the heating characteristics varied depending on the CNT content. When 0.1 wt% CNT was added, the maximum heating temperature reached 1055 °C at 300 s. However, specimens with 1 wt% and 10 wt% CNTs exhibited significantly shorter heating behavior, with lower heating temperatures of 500.2 and 458 °C, respectively.

**Figure 6:**
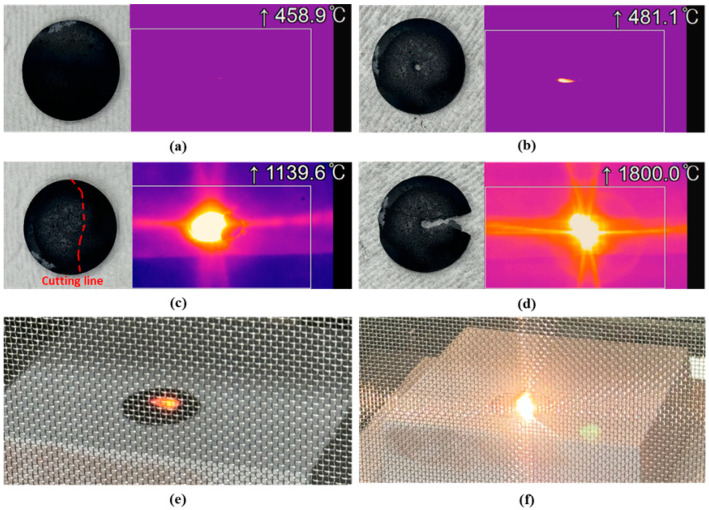
Microwave heating behavior of specimens under different post-processing conditions: (**a**) surface polishing, (**b**) central perforation, (**c**) midpoint fracture, (**d**) central perforation with partial material removal, (**e**) microwave heating behavior of a standard specimen, and (**f**) ultrahigh-temperature heating phenomenon (1800 °C) of specimen with central perforation and partial material removal.

**Figure 7:**
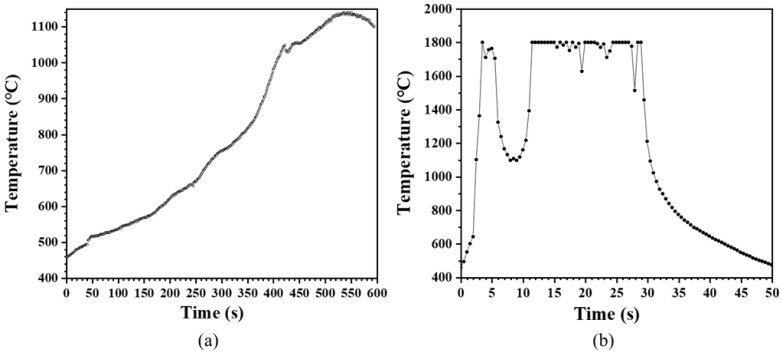
Microwave heating behavior based on specimen geometry: (**a**) specimen with midpoint fracture; (**b**) specimen with central perforation and partial material removal.

Specimens containing 10 wt% CNT exhibited variations in microwave-induced heating behavior depending on their geometry. To investigate this phenomenon further, additional processing was performed using different methods, followed by microwave heating tests. The corresponding results are presented in Figure 6. Specimens that underwent surface polishing or had a perforation at the center exhibited minimal heating. In contrast, specimens that were fractured at the center and then placed closely together demonstrated a gradual temperature increase, reaching 1141 °C after 10 min, as shown in Figure 7a. Notably, heating occurred only on one side of the specimen, with the fracture line serving as a boundary.

The specimen in which one direction was removed after perforation at the center exhibited an ultrahigh-temperature rapid heating phenomenon exceeding 1800 °C, as shown in Figure 7b. The actual heating temperature was expected to be even higher because of the temperature detection limit of the thermal imaging camera. Figure 6e presents the typical microwave-induced heating phenomenon observed in standard specimens, whereas Figure 6f shows the thermal imaging of the specimen that reached 1800 °C. Notably, the ultrahigh-temperature rapid heating at 1800 °C only occurred in localized, regions rather than across the entire specimen.

The ultrahigh-temperature rapid heating phenomenon, induced by the microwave irradiation of SiC and CNTs, is an unprecedented observation. Reproducibility tests conducted on identically fabricated specimens confirmed the recurrence of this phenomenon, particularly in the direction in which the material was removed, suggesting that it arose from an intrinsic mechanism.

SiC is a high-dielectric-loss material that undergoes rapid heating to temperatures exceeding 1000 °C within 1 min when exposed to a specific 2.45 GHz microwave frequency. The rapid heating of SiC under microwave irradiation has been attributed to the dipolar loss caused by the rotation and collision of dipoles within the material, as well as conduction loss, which results from electron migration and collision under an external electromagnetic field. Further studies are required to fully elucidate the action mechanism [23,24,25]. Attempts have been made to explain the observed phenomenon by applying established rapid heating mechanisms such as arc discharge [26] and flash sintering [27,28,29]. However, these explanations have been deemed inadequate to describe the unique characteristics of this phenomenon. To elucidate the underlying mechanism, further research will be conducted via a more detailed investigation into the effects of the CNT content and the precision of specimen processing methods. Through these studies, we aimed to systematically analyze and clarify the mechanism responsible for this unique rapid heating behavior.

## 4. Conclusions

The effect of CNT addition on PCS-based SiC was investigated. The specimens were fabricated by adjusting the CNT content in iodine-assisted mechanochemically cured PCS. As the CNT content increased, fine pores were reduced; however, at 10 wt% CNT, agglomerates were observed. The microwave heating performance varied with CNT content, with the highest heating temperature recorded at 0.1 wt% CNT. In contrast, at 1 wt% and 10 wt% CNT, the heating temperature decreased to approximately 500 °C. The post-processing of the specimens containing 10 wt% CNT resulted in distinct microwave heating behaviors. A specimen that was fractured at the midpoint and reassembled in proximity exhibited localized heating, reaching a maximum of 1140 °C on one side. Meanwhile, a specimen with central perforation and partial material removal exhibited an ultrahigh-temperature rapid heating phenomenon exceeding 1800 °C. This ultrahigh-temperature rapid heating effect induced by microwaves is unprecedented and has not been reported previously. Further studies considering detailed factors, such as specimen processing methods and CNT distribution, are required to elucidate the underlying mechanisms.

## Data Availability

The original contributions presented in this study are included in the article. Further inquiries can be directed to the corresponding author.
